# Rhizosphere bacterial carbon turnover is higher in nucleic acids than membrane lipids: implications for understanding soil carbon cycling

**DOI:** 10.3389/fmicb.2015.00268

**Published:** 2015-04-09

**Authors:** Ashish A. Malik, Helena Dannert, Robert I. Griffiths, Bruce C. Thomson, Gerd Gleixner

**Affiliations:** ^1^Department of Biogeochemical Processes, Max Planck Institute for BiogeochemistryJena, Germany; ^2^Centre for Ecology and HydrologyWallingford, UK

**Keywords:** ^13^C tracer experiment, bacteria, carbon, DNA, fungi, PLFA, RNA, soil

## Abstract

Using a pulse chase ^13^CO_2_ plant labeling experiment we compared the flow of plant carbon into macromolecular fractions of rhizosphere soil microorganisms. Time dependent ^13^C dilution patterns in microbial cellular fractions were used to calculate their turnover time. The turnover times of microbial biomolecules were found to vary: microbial RNA (19 h) and DNA (30 h) turned over fastest followed by chloroform fumigation extraction-derived soluble cell lysis products (14 days), while phospholipid fatty acids (PLFAs) had the slowest turnover (42 days). PLFA/NLFA ^13^C analyses suggest that both mutualistic arbuscular mycorrhizal and saprophytic fungi are dominant in initial plant carbon uptake. In contrast, high initial ^13^C enrichment in RNA hints at bacterial importance in initial C uptake due to the dominance of bacterial derived RNA in total extracts of soil RNA. To explain this discrepancy, we observed low renewal rate of bacterial lipids, which may therefore bias lipid fatty acid based interpretations of the role of bacteria in soil microbial food webs. Based on our findings, we question current assumptions regarding plant-microbe carbon flux and suggest that the rhizosphere bacterial contribution to plant assimilate uptake could be higher. This highlights the need for more detailed quantitative investigations with nucleic acid biomarkers to further validate these findings.

## Introduction

Soil microorganisms are a crucial link between the above and belowground components of ecosystems. Moreover, their role in cycling of soil organic matter (SOM) carbon highlights their importance in ecosystem functioning (Schimel and Schaeffer, 2012; Gleixner, 2013). However, despite the relatively large size and temporal sensitivity of the soil organic carbon pool and its importance in terrestrial ecosystem functioning (including maintenance of agricultural productivity and mitigation of atmospheric CO_2_ levels), our knowledge of the soil processes involved in the formation and decomposition of SOM remains very limited (Allison and Martiny, 2008; Lal, 2010). This has led to large uncertainty in how global soil carbon stocks will respond to climate warming (Canadell et al., 2007), primarily due to a lack of understanding of whether environmental change will lead to a reduction or increase in soil carbon through altered microbial functioning (Wieder et al., 2013). This warrants more investigations aiming at having a better mechanistic understanding of microbial processes contributing to the persistence and transformation of carbon in soil.

Plant organic matter constitutes the primary source of soil carbon. In addition to root exudation, plant inputs in the form of shoot and root litter provide a carbon source for soil organisms. Root-associated microbial food webs are not only linked to the flow of energy and nutrients between plants and soil organisms but also facilitate plant growth (Wardle et al., 2004; de Boer et al., 2006). Fungi and bacteria are the major decomposers of plant derived carbon and it is generally acknowledged that niche differentiation exists between bacterial and fungal decomposers with respect to substrate complexity (de Boer et al., 2006). Decomposition of labile substrates, such as root exudates is often attributed to bacteria particularly in the favorable, resource-rich rhizosphere environment (de Boer et al., 2005). Indeed, a variety of bacteria particularly from the Pseudomonadaceae or Burkholderiaceae families have been implicated in the rapid assimilation of root-derived plant carbon (Vandenkoornhuyse et al., 2007; Philippot et al., 2013). However, other studies have demonstrated a significant role of both symbiotic and saprophytic fungi in utilization of root derived carbon (Drigo et al., 2010; Balasooriya et al., 2014). Most significantly, arbuscular mycorrhizal fungi (AMF) have been linked to the rapid belowground translocation of plant carbon. It has been estimated that up to 20% of photoassimilates can be utilized by AM fungi, making mycorrhizal hyphal turnover a substantial process for carbon input into SOM (Bago et al., 2000; Godbold et al., 2006). These results point to a lack of consensual knowledge concerning microbial trophic interactions and the flow of carbon through the plant-rhizosphere-soil continuum. Therefore, a reassessment of the root associated microbial food web is warranted.

Soil microorganisms mineralize most newly fixed carbon by respiration but they also contribute to the maintenance of SOM through turnover and recycling of biomass (Gleixner et al., 2002; Simpson et al., 2007; Mambelli et al., 2011; Miltner et al., 2012; Throckmorton et al., 2012). The contribution of non-living microbial biomass to the formation of SOM is estimated to be as high as 80% of organic carbon in soil (Liang and Balser, 2011; Liang et al., 2011). Stabilization of certain molecules in soil is largely attributed to the molecular transformation or resynthesis of compounds by microorganisms to form products that appear to persist in soil (Kindler et al., 2006; Simpson et al., 2007; Gleixner, 2013). Although microbial derived carbon inputs to soil are now increasingly acknowledged as a major source of SOM, the contribution of different microbial compounds to soil carbon storage remains poorly understood. Variable turnover rates and consequent differing retention times of microbial cellular fractions in soil are thought to contribute differently to soil carbon storage. Therefore, there is a need to determine the relationship between microbial macromolecular structure and carbon turnover in soil.

The flow of carbon from plants into soil can be examined by ^13^CO_2_ pulse labeling of plants and subsequent isotope analysis of different carbon pools in the plant-soil system (Griffiths et al., 2004). The translocation of ^13^C through root exudates and fine root turnover into rhizosphere microorganisms can be traced by measuring the amount of ^13^C in microbial biomarkers, also enabling estimation of carbon turnover times (Ostle et al., 2003; Leake et al., 2006; Clayton et al., 2010). Due to technological developments in stable isotope analyses, it is now possible to measure ^13^C content in various biomarkers. The liquid chromatography–isotope ratio mass spectrometry (LC-IRMS) interface allows rapid and accurate compound specific isotope measurements in aqueous extracts (Malik et al., 2012; Scheibe et al., 2012). Additionally, measurement of ^13^C labeled plant released carbon permits the estimation of turnover rates for major microbial functional compounds such as genomic DNA, ribosomal RNA, and cell membrane lipid fatty acids.

We hypothesized that microbial macromolecular structure and its biochemical composition plays an important role in determining the degree of microbial biomass contribution to soil carbon formation, by affecting the carbon residence time. Therefore, we compared the turnover rates of different soil extracted microbial compounds following a plant pulse chase experiment. Stable isotope probing with a multi-biomarker approach was employed to reappraise soil trophic interactions in terms of carbon flow through the plant-rhizosphere-soil continuum. A greenhouse-based, pulse chase ^13^CO_2_ plant labeling experiment was performed in mesocosms with a mycorrhizal plant species and an inter-disciplinary analytical approach combining molecular biology and stable isotope analytical techniques were used to monitor carbon cycling in this system. Plant released ^13^C was traced into rhizosphere soil microbial DNA, RNA, fatty acids and extractable microbial biomass [chloroform fumigation extraction (CFE)-derived]. Additionally, C flow into soil microbial functional groups was assessed through phospholipid/neutral lipid fatty acid (PLFA/NLFA) and ribosomal RNA ^13^C analyses. Microbial carbon fluxes were also linked to plant and soil C pools in order to investigate belowground carbon dynamics and deduce a mechanistic understanding of carbon flow in plant–soil systems.

## Materials and Methods

### Plant and Soil System

Soil was collected from the Jena Biodiversity Experiment site located in Jena, Germany in September 2012 from an arable field (Roscher et al., 2004; Lange et al., 2014). Soil was sieved (<2 mm), all visible roots were removed and prior to establishing mesocosms homogenized soil was stored at 4°C. Polystyrene pots (1 L) were filled with 800 g of soil and incubated in the greenhouse (40 pots were destructively sampled on nine occasions). Mesocosms were uniformly watered with an automated irrigation system 2–3 times per day; and additional light (Son-T Agro 430 W HPS bulbs, primary light range = 520–610 nm, Philips Lighting Company, Somerset, NJ, USA) was provided 12 h per day. Experimental mesocosms were left to equilibrate for 2 weeks before sowing *Chenopodium ambrosioides*, a temperate herb that is known to form mycorrhizal association. After developing true leaves (4 weeks later), plants were thinned leaving three per mesocosm.

### ^13^CO_2_ Labeling

After 3 months of plant growth, plants were exposed to ^13^CO_2_ in a 2000 L air tight glass chamber. Thirty planted pots were introduced into the chamber (three control replicates were not subjected to the^13^CO_2_ pulse). Prior to pulsing, the chamber was flushed with CO_2_-free synthetic air until the concentration of CO_2_ fell below 50 ppm. Enriched ^13^CO_2_ (99 atom %) was introduced into the labeling chamber at a flow rate of 100 ml min^-1^ and was cycled throughout the chamber using an internal ventilation system to achieve uniform labeling. Photosynthetic uptake of CO_2_ was monitored using a Picarro 2101i (Picarro Inc., Santa Clara, CA, USA) and throughout the labeling period of 10 h, the CO_2_ concentration in the chamber was maintained between 350 and 400 ppm. Photosynthetic rate was around 100 ppm h^-1^ at the beginning and dropped below 50 ppm h^-1^ toward the end of the labeling period. At the end of the labeling period (10 h), the chamber was opened, and the plants were returned to the greenhouse.

### Sampling

Destructive sampling was performed at 1, 3, 12, and 24 h, then 2, 4, 7, 14, and 21 days after the pulse labeling. At each time point 3 mesocosms were sampled for rhizosphere soil and plant parts. After discarding the top 5 cm the rest of the soil was considered as rhizosphere soil, due to heavy colonization of roots. Soil was then sieved to <2 mm and fine roots were extensively removed by handpicking. An aliquot of soil was stored at -80°C prior to nucleic acids extraction. Additionally, soil for lipid and microbial biomass extractions was stored at -20°C. A smaller aliquot for ^13^C measurement of bulk SOM was dried at 40°C. Plant roots, stems, and leaves for ^13^C measurement were washed with water in order to remove adherent soil residues and dried at 40°C.

### ^13^C Analyses of Plant and Soil Organic Matter

Before^13^C analysis of bulk soil organic carbon, dried soil was ground using a ball-mill and acidified to remove carbonates. After drying, plant parts were shredded and ground in a ball-mill (Kramer and Gleixner, 2008). Bulk ^13^C analysis of SOM and plant material was performed on an elemental analyzer coupled to an isotope ratio mass spectrometer (EA model CE 1100 coupled on-line via a Con Flo III[27] interface with a Delta^+^ isotope ratio mass spectrometer; all supplied by Thermo Fisher Scientific, Germany). To measure the plant and soil respired ^13^CO_2_, three plant pots were placed into a 2000 L airtight glass chamber in the dark (post sunset) at 1, 2, 4, 7, 14, and 21 days after pulse labeling of plants. The concentration of CO_2_ in the chamber and its ^13^C content was continuously monitored for 90 min at each time point using a Picarro 2101i (Picarro Inc., Santa Clara, CA, USA). To estimate the δ^13^C value of total respired CO_2_, the reciprocal of CO_2_ concentration was plotted against δ^13^C value of chamber CO_2_ over the analysis period (Keeling plot), with the y-intercept representing the δ^13^C value of total respired CO_2_ (Pataki et al., 2003).

### ^13^C Analysis of Microbial Biomass and Respired CO_2_

Frozen soil (1 g) was placed in a 10 mL glass vial, thawed and incubated for approximately 24 h in the dark at room temperature. Microbially respired ^13^CO_2_ collected in the headspace was measured using a gas chromatography isotope ratio mass spectrometer (GC-IRMS, Delta^+^ XL, Thermo Fisher Scientific, Germany) coupled to a PAL-autosampler (CTC Analytics) with general purpose (GP) interface (Thermo Fisher Scientific, Germany). Microbial biomass from soil was obtained using a slightly modified version of the CFE method (Vance et al., 1987; Malik et al., 2013). Soil (7 g wet weight) was fumigated with chloroform gas and organic carbon was extracted from both fumigated and non-fumigated soils. Soil extracts were analyzed in the bulk (μEA) mode on an HPLC-IRMS (HPLC system coupled to a Delta^+^ XP IRMS through an LC IsoLink interface; Thermo Fisher Scientific, Germany).

### ^13^C Analysis of Microbial Nucleic Acids

Microbial nucleic acids (DNA and RNA) were extracted from 0.5 g soil using a previously described method (Griffiths et al., 2000) including a double bead beating to improve the yield of nucleic acids. DNA and RNA were then purified and separated using an All Prep DNA/RNA mini kit (Qiagen, Germany) according to the manufacturer’s instructions. Following elution in molecular grade water, the purity and concentration of DNA and RNA were assessed by gel electrophoresis and Nanodrop quantification (NanoDrop 8000 UV-Vis Spectrophotometer, UK). The purity of total RNA was also assessed using an Agilent TapeStation using a R6K ScreenTape (Agilent Technologies, UK) which provides improved size resolution compared to agarose gel electrophoresis. ^13^C analysis of DNA and RNA was performed on an HPLC-IRMS system in SEC mode (Malik et al., 2012). SEC was performed on a mixed bed analytical column (TSK-GEL GMPWXL- 7.8 mm × 30 cm; Tosoh Bioscience, Germany) with a mobile phase of 20 mM phosphate buffer (pH 6.2) at a flow rate of 500 μL min^-1^. This method is an improvement over previously described methods (Manefield et al., 2002; Miyatake et al., 2009; Thomson et al., 2013) as nucleic acids can be measured directly without any pre-processing. Moreover, online SEC-based separation of protocol contaminants as well as soil co-extracts from nucleic acids greatly improves the accuracy and precision of measurement. Samples (50 μL containing 100–600 ng DNA/RNA) were manually injected into the system. Subsequently, DNA or RNA chromatogram peaks were identified by correlating their retention times against standard yeast rRNA (Sigma-Aldrich, Germany), calf thymus DNA (Trevigen, USA), and RNA size ladder (Invitrogen-Life technologies, Germany).

### Lipid Biomarker ^13^C Analysis

Microbial lipids were extracted from ∼50 g (dry weight) of soil according to a modified Bligh and Dyer extraction protocol (Bligh and Dyer, 1959; Kramer and Gleixner, 2008). Extractions were carried out using a mixture of chloroform (CHCl_3_), methanol (MeOH), and 0.05 M phosphate buffer (pH 7.4; 1:2:0.8 v:v:v). Extracted lipids were separated into neutral lipids, phospholipids and glycolipids using silica columns. Fatty acid methyl esters (FAMEs) were then isolated by mild alkaline hydrolysis and methylation of fatty acids, followed by the removal of unsubstituted FAMEs. PLFAs were then separated into saturated fatty acids (SATFAs), monounsaturated fatty acids (MUFAs), and polyunsaturated fatty acids (PUFAs). All extracts were dried under a nitrogen stream, resuspended in a 200 μL stock solution containing n19:0 in isooctane as internal standard. Gas chromatography flame ionization detector (GC-FID, Hewlett Packard HP 6890 series GC-System coupled with a FID; Agilent Technologies, Palo Alto, CA, USA) was used to quantify the PLFA content. ^13^C analysis was performed using gas chromatography-combustion-isotope ratio mass spectrometry (GC-C-IRMS: HP5890 GC, Agilent Technologies, Palo Alto, CA, USA; connected to IRMS Deltaplus XL, Finnigan MAT, Bremen, Germany; via a combustion interface GC Combustion III Finnigan MAT, Bremen, Germany).

### Isotope Expression

The net^13^C enrichment in various carbon pools in the plant–soil system was estimated as Δδ^13^C value which denotes the change in δ^13^C value post labeling relative to the unlabeled control. We also calculated the absolute amounts of ^13^C incorporated into different biomarkers which is expressed in pg ^13^C g^-1^ of soil (Boschker et al., 1998). Thus, the difference (Δ*δ*^13^C) in the *δ*^13^C values between labeled and unlabeled as well as the ^13^C content in individual biomolecules per g of soil will be used throughout the results section.

### Calculating Turnover Time

Empirical C turnover time (synonymously referred to as mean residence time) of microbial fractions was obtained by estimating the pulse ^13^C dilution rate in individual fractions using a negative exponential model.

F*=F*e−kt0

where *F*_0_ and F^∗^ is the net ^13^C enrichment at time t 0 and time t; and k is the rate constant (Supplementary Figure S1). Turnover time was calculated as inverse of the ^13^C dilution rate constant *k* (Ostle et al., 2003; Butler et al., 2004).

## Results

### ^13^C in respired CO_2_, Roots and Soil

Following pulse labeling, ^13^C fate was assessed into belowground plant material as well as in total respired CO_2_ (Supplementary Figure S2). The Δδ^13^C of plant and soil respired CO_2_ was very high (3828.8 ‰) 1 day after the pulse, then dropped drastically over the following 3 days (891.4 ‰ on day 4) and finally reached a stationary phase toward the end of the experiment (159.6 ‰ after 3 weeks). The Δδ^13^C of primary roots at 1 day and 3 weeks after the pulse event was 400.9 ± 241.5‰ and 307.8 ± 123.4‰, compared to fine roots which had a Δδ^13^C of 326.2 ± 72.9‰ and 158.7 ± 55.7‰, respectively. Interestingly, the decrease in ^13^C enrichment in primary roots over the experimental period was less than that of fine roots. The incorporation of ^13^C into SOM was minor, with a mean Δδ^13^C value of 2.7‰ throughout the experiment (Supplementary Figure S3). However, there was a significant increase between 1 and 4 days after pulse labeling.

### ^13^C in Microbially Respired CO_2_ and Microbial Biomass

**FIGURE 1:**
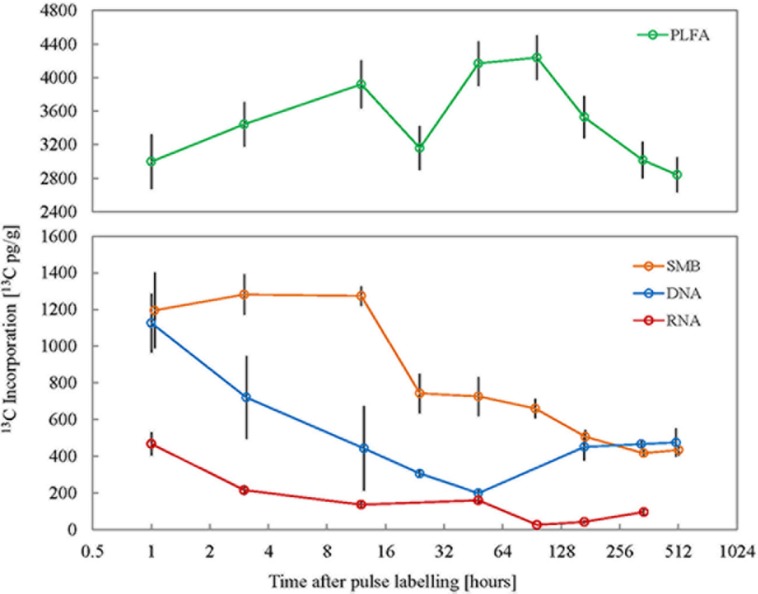
**Isotope enrichment in different microbial cellular fractions (*n* = 3) over the experimental period following ^13^CO_2_ pulse labeling of plants.** SMB: total microbial biomass obtained from chloroform fumigation extraction, PLFA: sum of all individual quantifiable PLFAs, RNA: total ribonucleic acid, DNA: total deoxyribonucleic acids. X-axis represents time post the 10 h pulse labeling period on a log_2_ scale and error bars represent standard error.

The source of microbially respired CO_2_ was discerned through its pulse ^13^C enrichment, which reflected the enrichment in the plant pools_._ The Δδ^13^C of microbially respired CO_2_ was highest (231.5 ± 83.5‰) immediately after the pulse, and then dropped gradually over the following weeks to finally reach a value of 23.2 ± 15.3‰ at 3 week after pulse labeling (Supplementary Figure S4). The soil microbial biomass (SMB) carbon content exhibited only minor variations across all time points suggesting a steady state, showing that the microbial biomass was constant throughout the experiment. When used in combination with stable carbon isotope analysis the CFE product can be used to track the source of microbial carbon. ^13^C enrichment in SMB was highest immediately after the pulse labeling of plants and remained so for at least 12 h after the pulse (**Figure 1**). The mean Δδ^13^C of SMB 1, 3, and 12 h after pulse labeling was 95.5 ± 28.6, 102.4 ± 15.3, and 101.8 ± 7.3‰; and the corresponding mean ^13^C enrichment was 1195.6 ± 357, 1281.7 ± 191.6, and 1273.7 ± 91.4 pg^13^C g^-1^ soil, respectively. This decreased to 59.3 ± 14.9‰ (742.8 ± 186.5 pg^13^C g^-1^) at 24 h after pulse labeling and by the final sampling point 3 weeks after pulse labeling; the Δδ^13^C had steadily decreased to 34.7 ± 4.1‰ (416.5 ± 34.9 pg^13^C g^-1^). Empirical C turnover time of CFE microbial biomass was estimated to be 14 days.

### ^13^C in Microbial DNA and RNA

The incorporation of ^13^C into DNA and RNA was also measured to assess the turnover of these compounds in soil microorganisms. Soil microbial total DNA and RNA both showed highest ^13^C enrichment immediately after pulse labeling of plants (**Figure 1**). The mean Δδ^13^C of DNA 1 h after pulse labeling was 37.7 ± 9.3‰ (1126.5 ± 278.3 pg^13^C g^-1^) and decreased to its lowest at 6.7 ± 0.9‰ (198.7 ± 26.8 pg^13^C g^-1^) 48 h after the pulse event. This was followed by a minor increase to ∼15‰ (464 pg^13^C g^-1^) throughout the remainder of the experiment. The ^13^C enrichment in total RNA was also highest immediately after the pulse labeling event; 1 h after the pulse mean Δδ^13^C of RNA was 66.2 ± 15.6‰ (467.5 ± 110 pg^13^C g^-1^). This gradually decreased over subsequent sampling points to the lowest level of enrichment (3.7 ± 1.1‰; 25.9 ± 4.8 pg^13^C g^-1^) 4 days after labeling. However, 2 weeks after labeling there was a significant increase in the amount of ^13^C in RNA (13.6 ± 3.6‰; 95.8 ± 24.9 pg^13^C g^-1^) and this persisted until the final sampling point. Although the Δ*δ*^13^C of RNA was higher than that of the DNA in the initial experimental phase after the pulse event, the mean amount of ^13^C in RNA per unit soil was lower compared to DNA. This was because the amount of extractable RNA (1.6 μg g^-1^) was about four times lower than that of DNA (6.7 μg g^-1^) in the soil type used. The turnover time of soil microbial RNA and DNA was estimated at 19 and 30 h, respectively.

### ^13^C in PLFA

**FIGURE 2:**
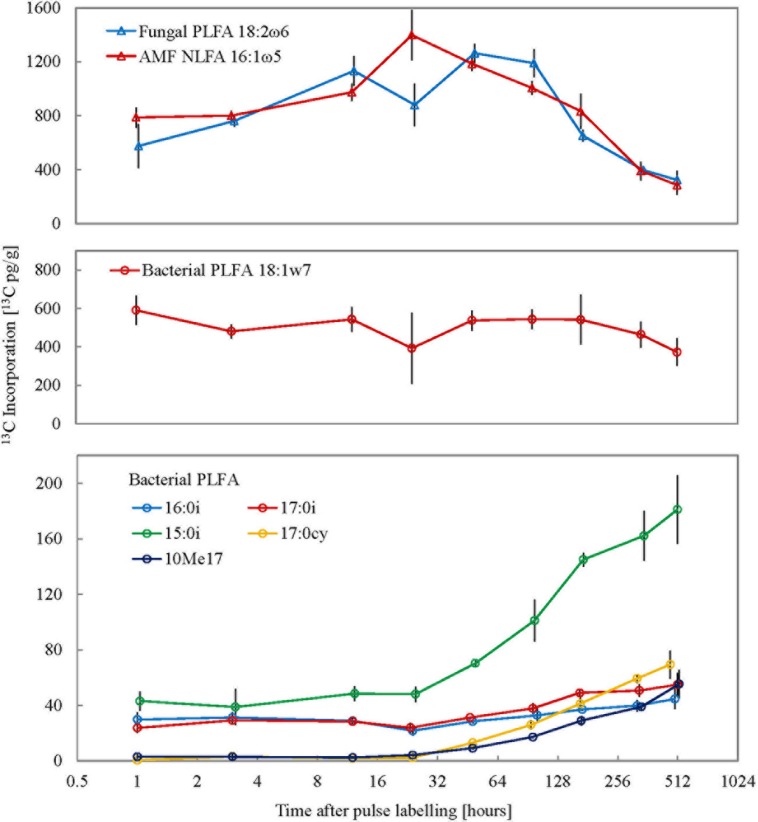
**Isotope enrichment in different fungal and bacterial PLFA/NLFA markers (*n* = 3) over the time series.** *X*-axis represents time post the 10 h pulse labeling period on a log_2_ scale and error bars represent SE.

Corroborating the consistent microbial biomass amounts measured with CFE, the abundances of total PLFAs and individual markers were consistent with only minor variation throughout the experiment (data not shown). After pulse labeling of plants ^13^C incorporation into total soil extractable PLFA remained largely similar throughout the experimental period (average Δδ^13^C = 41.1 ± 9.1‰; average ^13^C incorporation = 3478.2 ± 524.6 pg^13^C g^-1^; **Figure 1**). To calculate turnover time only the decay following peak ^13^C enrichment was used (Supplementary Figure S1). Therefore, the empirical C turnover time of total PLFAs at 42 days is only a rough estimation. The ^13^C incorporation patterns over time in different PLFA biomarkers were shown to be highly variable (**Figure 2**). Incorporation of ^13^C was on average an order of magnitude higher in fungal PLFAs compared to bacterial ones with the exception of the Gram negative PLFA 18:1ω7 marker which had high enrichment (**Figure 2**). Highest enrichment was observed in the fungal fatty acids, PLFA 18:2ω6 and NLFA 16:1ω5 had a mean Δδ^13^C of 409.3 ± 173.8‰ (796.2 ± 344 pg^13^C g^-1^) and 100.1 ± 73‰ (850.1 ± 351.6 pg^13^C g^-1^), respectively. The ^13^C enrichment in the AMF NLFA marker 16:1ω5 peaked 24 h after the pulse (1397.2 ± 322.5 pg^13^C g^-1^). The enrichment in the AMF marker decreased gradually over time to a minimum of 285 ± 124.1‰ 3 weeks after labeling. Contrastingly, ^13^C enrichment of different bacterial PLFAs showed a divergent temporal pattern with roughly three discernable trends: (1) high enrichment immediately after the pulse event that remained constant over time followed by a small drop after a week, e.g., PLFA 18:1ω7; (2) consistent medium to low enrichment shortly after the pulse with a gradual increase after 4 days, e.g., PLFAs 15:0i, 16:0i, 17:0a, 17:0i; and (3) negligible enrichment immediately after the pulse followed by a small gradual increase toward the end of the experimental period, e.g., PLFAs 17:0cy, 10Me17. The average ^13^C incorporation in representative Gram negative (18:1ω7) and Gram positive (16:0i) PLFA markers across all time points was 495.8 ± 74.5 pg^13^C g^-1^ and 32.6 ± 6.9 pg^13^C g^-1^, respectively. The ^13^C enrichment in the Actinomycetes biomarker PLFA 10Me17 was negligible post-labeling and gradually increased throughout the sampling regime to reach the highest value of 55.2 ± 17.8 pg^13^C g^-1^ after 3 weeks. Finally, total bacterial ^13^C enrichment showed a late increase toward the end of the experimental period (Supplementary Figure S5).

## Discussion

This study used a novel approach combining stable isotope techniques and molecular biology to better understand C cycling in grassland systems. Here we have traced ^13^CO_2_ into plants roots and subsequently into soil microbial communities. Pulse ^13^CO_2_ fixed by plants was found to be differentially allocated to different plant parts and was either used for respiration, biomass formation and/or storage. Up to 2 days after the pulse was applied, total respired CO_2_ was highly enriched in ^13^C indicative of fast respiratory fluxes. Although we did not analyze root exudates, based on the respiratory pattern we expected that the pulse ^13^C would be rapidly translocated into the rhizosphere as labile substances in the form of root exudates, which would serve as rhizodeposited C sources for rhizosphere microorganisms. As a consequence of this dependence, the ^13^C incorporation patterns in the rhizosphere soil microbial compounds that are turned over faster reflect those of the labile plant pools. Due to the rapid growth and multiplication of microorganisms,^13^C from the pulse event was immediately incorporated into all microbial fractions; however, variable decomposition kinetics were observed leading to variable carbon turnover time estimates (**Table 1**). The initial high ^13^C enrichment in all fractions suggests active production of all biosynthetic compounds despite a variable overall carbon turnover time for each fraction. A mixed signal was obtained due to the presence of slow and fast turnover substrate pools as well as due to the different carbon use strategies of saprophytic and mutualistic microbial groups; the former decompose complex organic matter whereas the latter depend on plant root exudates for carbon. The soil mesocosms used in the experiment were extensively colonized by roots, therefore it was expected that indicators of non-rhizosphere bulk soil microorganism would be either absent or negligible. The scope of this study was to investigate the carbon flow in bacteria, fungi and other microeukaryotes only, as sieving of soil excludes other eukaryotes.

**Table 1:** Comparison of the turnover time of different cellular fractions from rhizosphere microorganisms.

Microbial biochemical fraction	Turnover time
Ribonucleic acids (RNA)	0.8 days (19 h)
Deoxyribonucleic acids (DNA)	1.3 days (30 h)
Total CFE microbial biomass	14 days
Phospholipid fatty acids (PLFA)	42 days

Microbial biomass extracted by the widely used CFE procedure represented cell lysis soluble extracts (Franzluebbers, 1999; Philippot et al., 2012) and had a much slower carbon turnover. Phospholipid fatty acids (PLFAs) which form part of microbial cell membranes are steadily produced during cell growth and multiplication and therefore have a relatively slow carbon turnover compared to other cellular macromolecules. In spite of this, PLFAs had the largest ^13^C enrichment per gram of soil which is likely due to its larger pool size. Higher ^13^C enrichment was observed in DNA and RNA immediately after the pulse, and this decreased rapidly, suggestive of higher carbon turnover rates. Similar turnover times have been previously reported (Ostle et al., 2003; Rousk and Bååth, 2011; Balasooriya et al., 2014). As expected, DNA shows slower carbon turnover compared to RNA (Manefield et al., 2002; Radajewski et al., 2003; Dumont et al., 2011). This is because DNA is only renewed during cell multiplication and since DNA replication is semiconservative only half of the DNA is renewed (Meselson and Stahl, 1958). Higher carbon turnover in RNA is reflective of the active anabolic function of the macromolecule; also its production is independent of cell replication. However, higher ^13^C incorporation amount was observed in DNA compared to RNA which is due to the higher DNA pool size of soil microorganisms. Interestingly; ^13^C incorporation in both DNA and RNA remained high toward the end of the study. Such long term persistence of isotope tracer in soil nucleic acids has previously been observed in other field based pulse labeling experiments (Griffiths et al., 2006) and is an unexplained phenomenon at odds with the fast uptake typically observed. Sustained ^13^C persistence in nucleic acids could indicate cross-feeding amongst soil organisms or delayed release of labeled plant substrates. However, it could also suggest microbial carbon recycling. In nutrient-limited environments, microorganisms often employ the salvage pathway of nucleotide synthesis from nucleotide breakdown products rather than the *de novo* synthesis (Nyhan, 2005). An indication that soil microorganisms preferably use the former pathway to recycle nucleotides from previously degraded RNA is available from the late increase in ^13^C enrichment in RNA toward the end of the time series period (Dumont et al., 2011).

Microbial turnover in soil is a significant source for the maintenance of the SOM. In this experiment, the initial ^13^C dilution rate or decrease in ^13^C enrichment in all microbial biomarkers except fatty acids coincided with a small yet significant increase in ^13^C in bulk SOM. This confirms that microbial biomass contributes to SOM genesis through the iterative process of microbial metabolism, growth, multiplication and death (Torn et al., 1997; Miltner et al., 2012).

Correlation of the carbon sources of microbially respired CO_2_ with the biomass fractions could be used to assess the coupling between microbial respiration and biosynthesis (Supplementary Figure S6). CFE derived microbial biomass correlated best with microbial respiration followed by RNA and then DNA. This suggests that the CFE product is likely labile soluble cellular material that is linked to microbial metabolism. However, CFE microbial biomass turnover time was relatively high. These observations suggest that microbial biomass derived from the CFE procedure is a mixture of varied compounds differing in complexity and stability which warrants its detailed molecular characterization. RNA production is an energetically expensive anabolic process that is also closely linked to the microbial respiratory flux. There was no correlation between PLFA ^13^C enrichment and the amount of ^13^C in microbially respired CO_2_ suggesting that lipid turnover is very slow and that soil microorganisms generally have lower lipid fatty acid renewal.

Phospholipid fatty acid and NLFA markers when combined with carbon stable isotope analysis can be used to track the flow of labeled substrate into different functional groups of microorganisms (Boschker et al., 1998). High ^13^C incorporation in the AMF represented by the NLFA 16:1ω5 marker was observed immediately following the pulse which indicates that the AMF are important in the initial carbon transfer from plants into soil microorganisms (Treonis et al., 2004; Olsson and Johnson, 2005; Denef et al., 2007; Drigo et al., 2010; Balasooriya et al., 2014). The AMF form a symbiotic association with plant roots and depend on root exudates for their carbon requirement. The general fungal marker PLFA 18:2ω6 also had high ^13^C incorporation shortly after the pulse but peaked later in the experiment; this lag shows that saprophytic fungi depend on complex organic matter from plants, such as fine root litter but also labile root exudates. The decline in ^13^C content of this fungal PLFA marker correlates with an increase in most bacterial markers suggestive of a carbon transfer from fungi to bacteria (Drigo et al., 2010; Balasooriya et al., 2014). However, different bacterial markers showed divergent patterns in ^13^C incorporation highlighting the diversity of bacterial carbon use strategies (Vandenkoornhuyse et al., 2007; Kramer and Gleixner, 2008; Philippot et al., 2013). As a result, two ecological statuses of soil bacteria can be discerned based on their C substrate utilization: the mutualistic rhizosphere bacteria mostly Gram negative types that rely on plant root exudates; and the saprophytes mostly Gram positive bacteria including Actinomycetes, which feed on complex organic matter from plants and/or other microorganisms. However, it should be noted that these differences in carbon turnover could also arise due to variable turnover of PLFAs in different bacteria (Ratledge and Wilkinson, 1989). Gram positive bacterial cell envelopes are thicker and more complex which could lead to a slower turnover of PLFA markers representative of this bacterial group. Therefore this factor should be taken into consideration when interpreting results from PLFA stable isotope probing.

**FIGURE 3:**
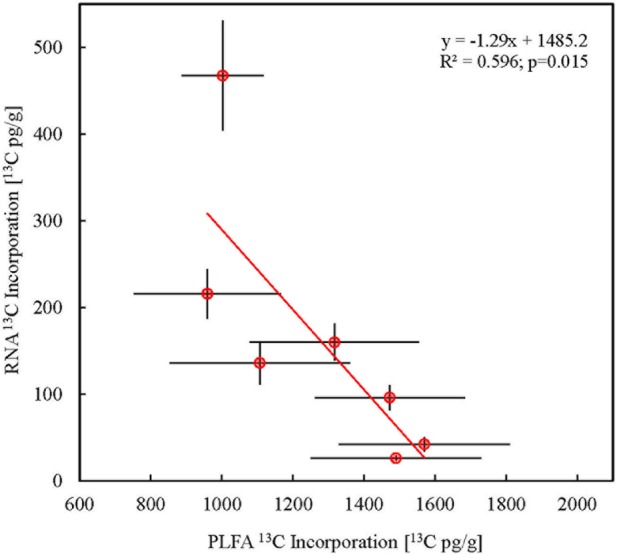
**Discrepancy in isotope enrichment in total bacterial PLFA and RNA (*n* = 3).** ^13^C incorporation in total bacterial PLFA was derived as the sum of ^13^C incorporation in all individual quantifiable bacterial PLFAs. Error bars represent SE.

Most RNA from the total RNA pool of soil microorganism was of bacterial origin (Urich et al., 2008). A high resolution gel image (Agilent TapeStation) showed no soil RNA bands which corresponded to purified large and small eukaryotic rRNA subunits (Supplementary Figure S7). Therefore, we considered total soil RNA to be a bacterial biomarker in order to monitor tracer carbon assimilation, assuming absence of eukaryotic/fungal RNA (Feinstein et al., 2009). The patterns of ^13^C incorporation in total microbial ribosomal RNA (**Figure 3**) thus signify that bacteria are also important utilizers of the initial plant C released into the rhizosphere. This is in contrast to most existing literature based on PLFA stable isotope probing which typically shows only a minor role of bacteria in initial plant carbon allocation compared with fungi (Olsson and Johnson, 2005; Drigo et al., 2010; Bahn et al., 2013; Balasooriya et al., 2014). Our data suggests that the bacterial contribution to plant assimilate uptake could be higher than previously estimated, and that PLFA based analysis tends to underestimate bacterial importance possibly due to the slow turnover of its cell wall lipids. This suggests that PLFA biomarkers may be less appropriate for monitoring fast carbon pulses that are often applied to study carbon fluxes in the rhizosphere. Moreover, we highlight the difficulties in comparing time series isotope enrichment in different biomarkers with inherently different turnover rates (Wolfe and Chinkes, 2005).

In summary, the estimated turnover rates of microbial components based on ^13^C incorporation patterns were found to significantly differ. The different degree of retention of microbial cellular fractions may imply their differential contribution to soil C storage. Thus microbial macromolecular structure is important in determining the degree of microbial contribution to SOM formation. Based on the results of our multi-biomarker tracer study we suggest there is a continuous recycling of carbon in the “soil microbial carbon loop” that could be an additive effect of exchanges within and between trophic levels. However, we question whether the current assumptions regarding plant–microbe C fluxes based on PLFA analyses and suggest that the contribution of rhizosphere bacteria to the initial conduit of plant-released C could be higher. We recommend more detailed quantitative investigations with nucleic acid biomarkers to better discern soil microbial trophic interactions.

## Author Contributions

AAM and GG designed research; AAM and HD performed the experiment, analyses and data acquisition; AAM drafted the manuscript; RIG, BCT, and GG contributed new reagents and analytical tools and were involved in critical revision and approval of the final version.

## Conflict of Interest Statement

The authors declare that the research was conducted in the absence of any commercial or financial relationships that could be construed as a potential conflict of interest.
