# Supplementary material for: Rhizosphere bacterial carbon turnover is higher in nucleic acids than membrane lipids: implications for understanding soil carbon cycling
Source: Front Microbiol. 2015 Apr 9;6:268. doi: 10.3389/fmicb.2015.00268 (PMC4391234; doi:10.3389/fmicb.2015.00268)
Supplement: Supplementary file 1 [file image_1.pdf]

Supporting Information Figure S1: ^{13}C enrichment trend in the microbial fractions fitted to an exponential decay function (only the highest to the lowest values were fitted to the function).

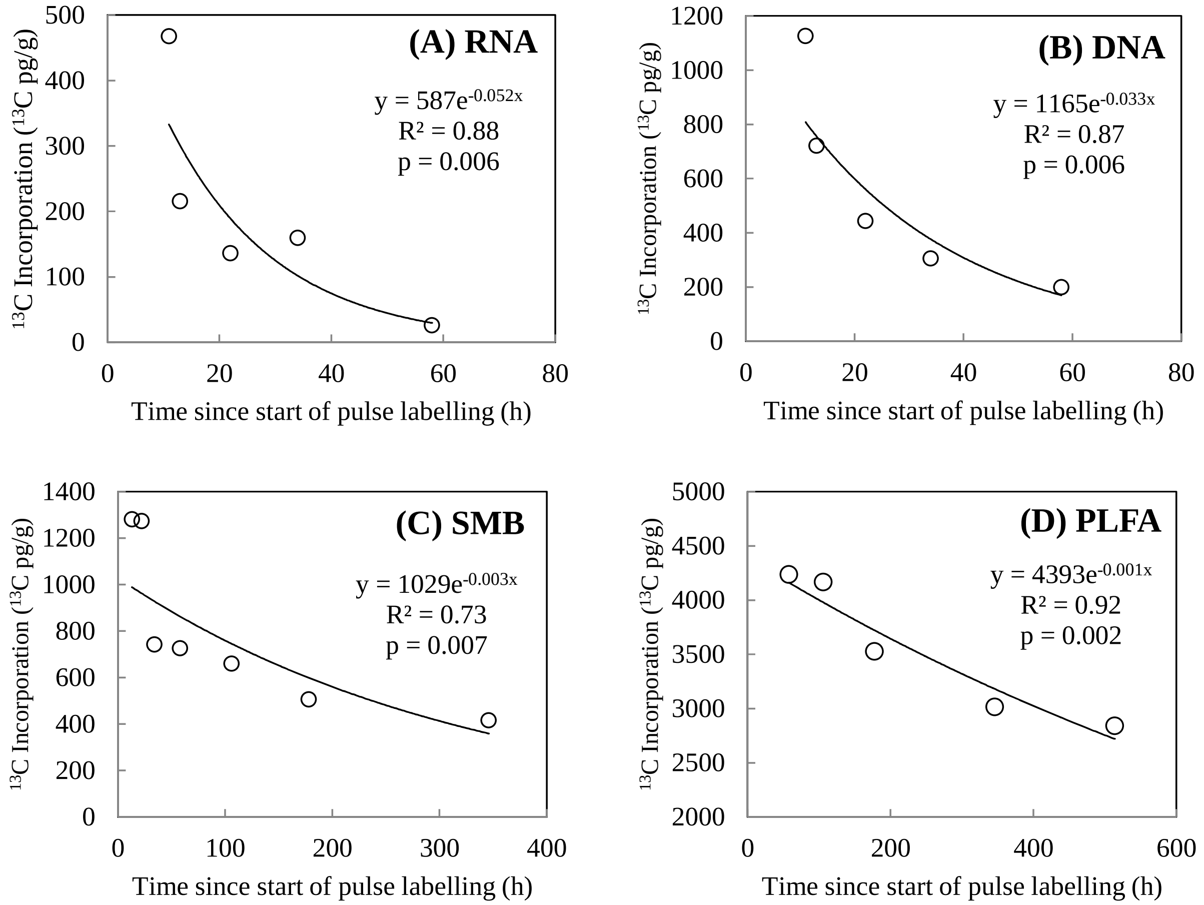

Supporting Information Figure S2(A-B): Isotope enrichment in different plant pools. X-axis represents time after end of pulse labelling and error bars represent standard error.

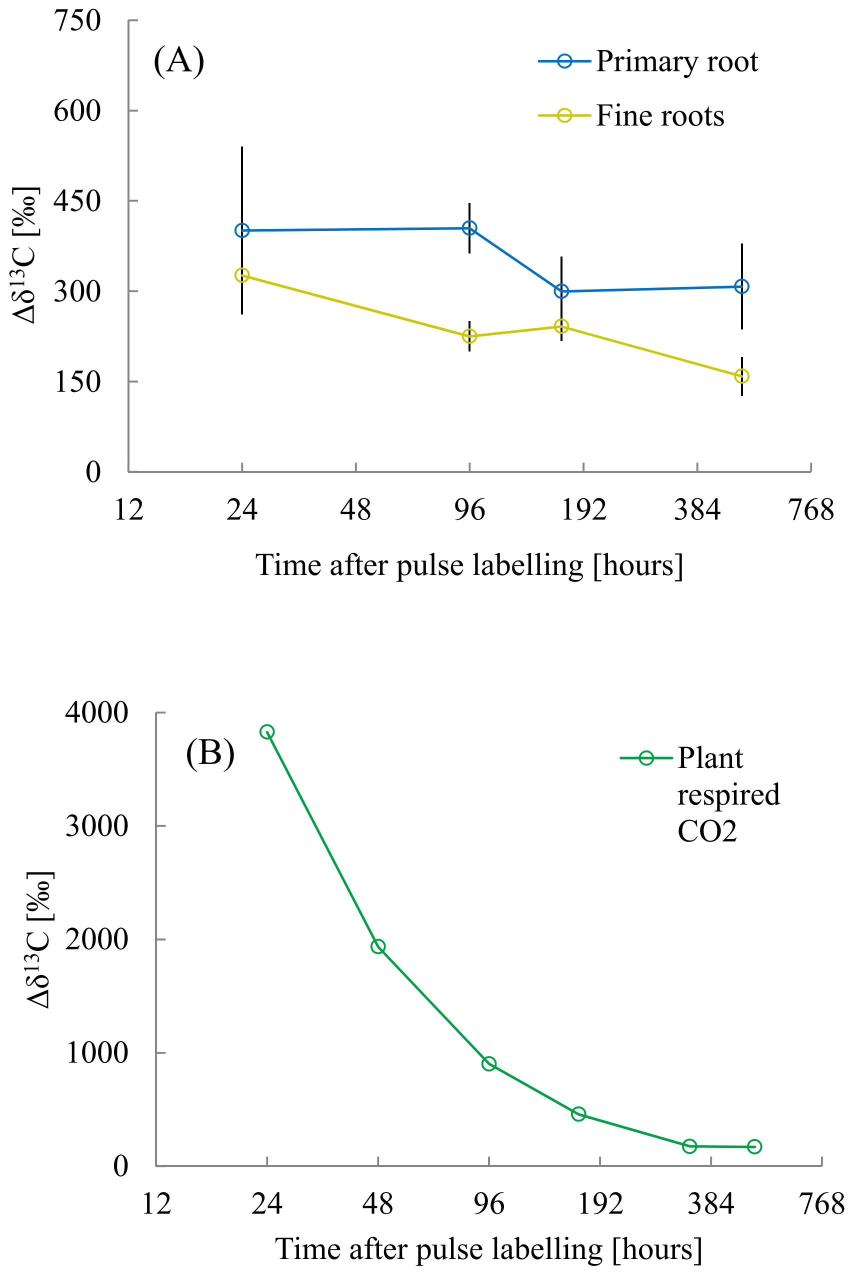

Supporting Information Figure S3: Isotope enrichment in bulk soil organic matter . X-axis represents time after end of pulse labelling and error bars represent standard error.

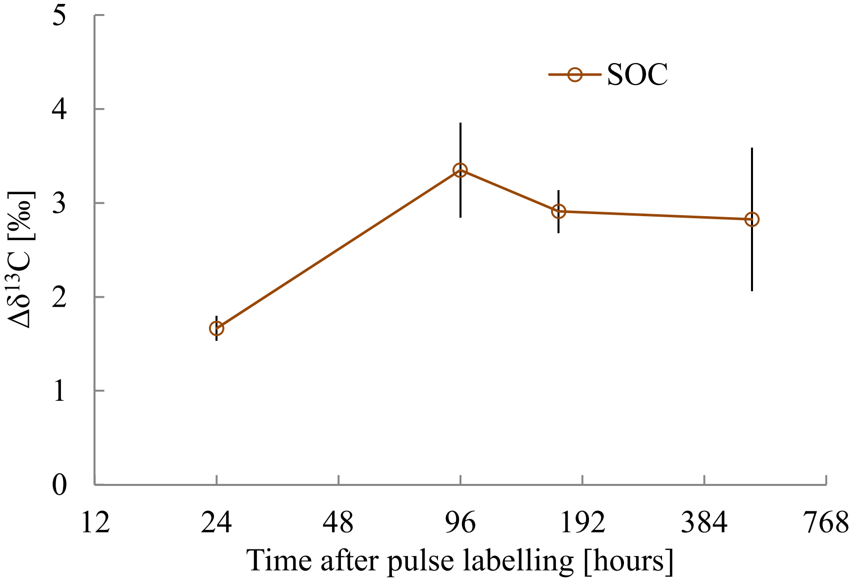

Supporting Information Figure S4: Isotope enrichment in microbially respired CO_2 (n=3). X-axis represents time after end of pulse labelling and error bars represent standard error. Note: the third time point in the time series does that follow the general decay fit which coul be because this was the only sampling point in the early morning (12 h after the pulse labelling).

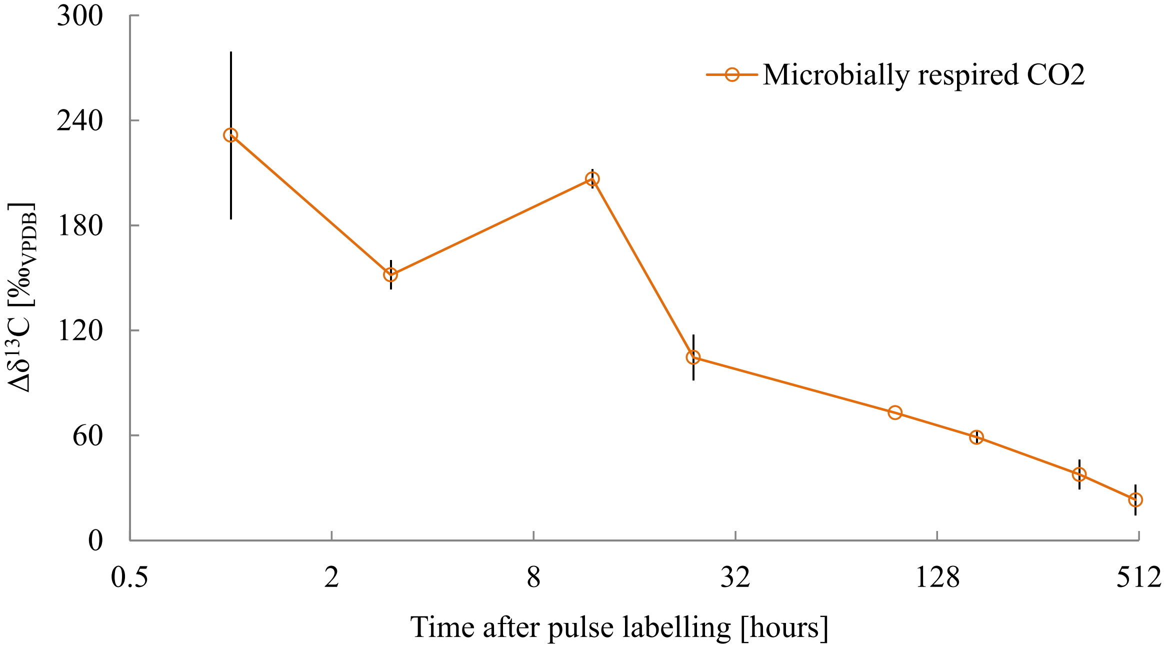

Supporting Information Figure S5: Isotope enrichment in total bacterial PLFA and RNA (n=3) over the time series. ^{13}C incorporation in total bacterial PLFA was derived as the sum of ^{13}C incorporation in all individual quantifiable bacterial PLFAs. X-axis represents time post the 10 h pulse labeling period and error bars represent standard error.

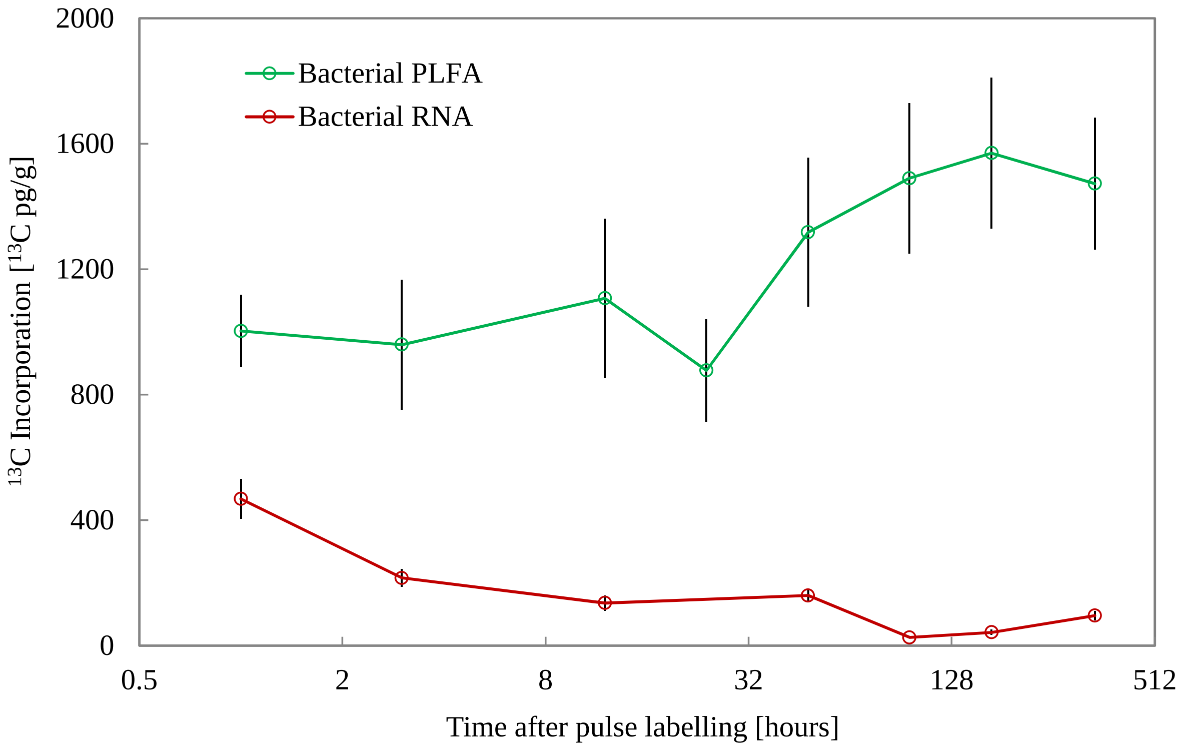

Supporting Information Figure S6: Correlation of ^{13}C enrichment in microbially respired CO_2 with different microbial fractions. A 1:1 dotted line is also shown in the figure. A slope closest to one means a good coupling between microbial biosynthesis and the respiratory flux.

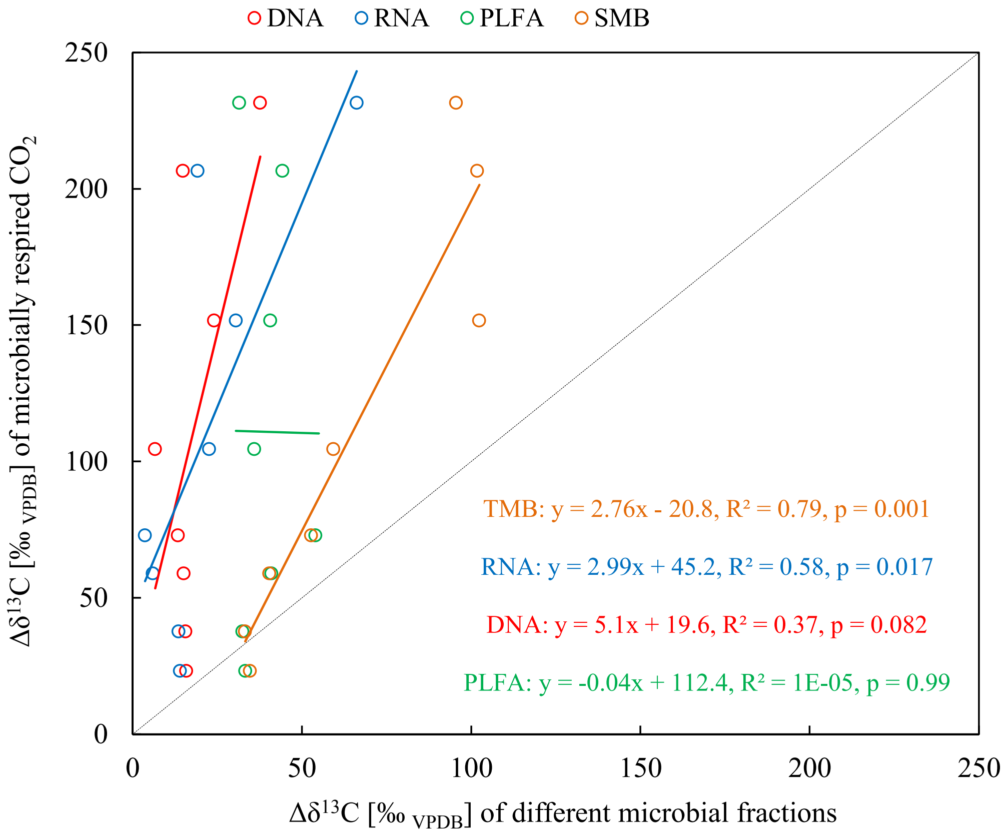

Supporting Information Figure S7: Size-based separation of RNA from soil microbial communities and pure cultures of E. coli and yeast on Agilent TapeStation-R6K ScreenTape. “Soil RNA EUB 338” is the captured 16S rRNA from the soil total RNA pool using magnetic bead capture hybridization (Miyatake et al., 2009). EUB 338 was the universal eubacterial probe used. E. coli and yeast were cultured in LB broth and RNA extracted using the standard phenol-chloroform extraction protocol (Griffiths et al., 2010).

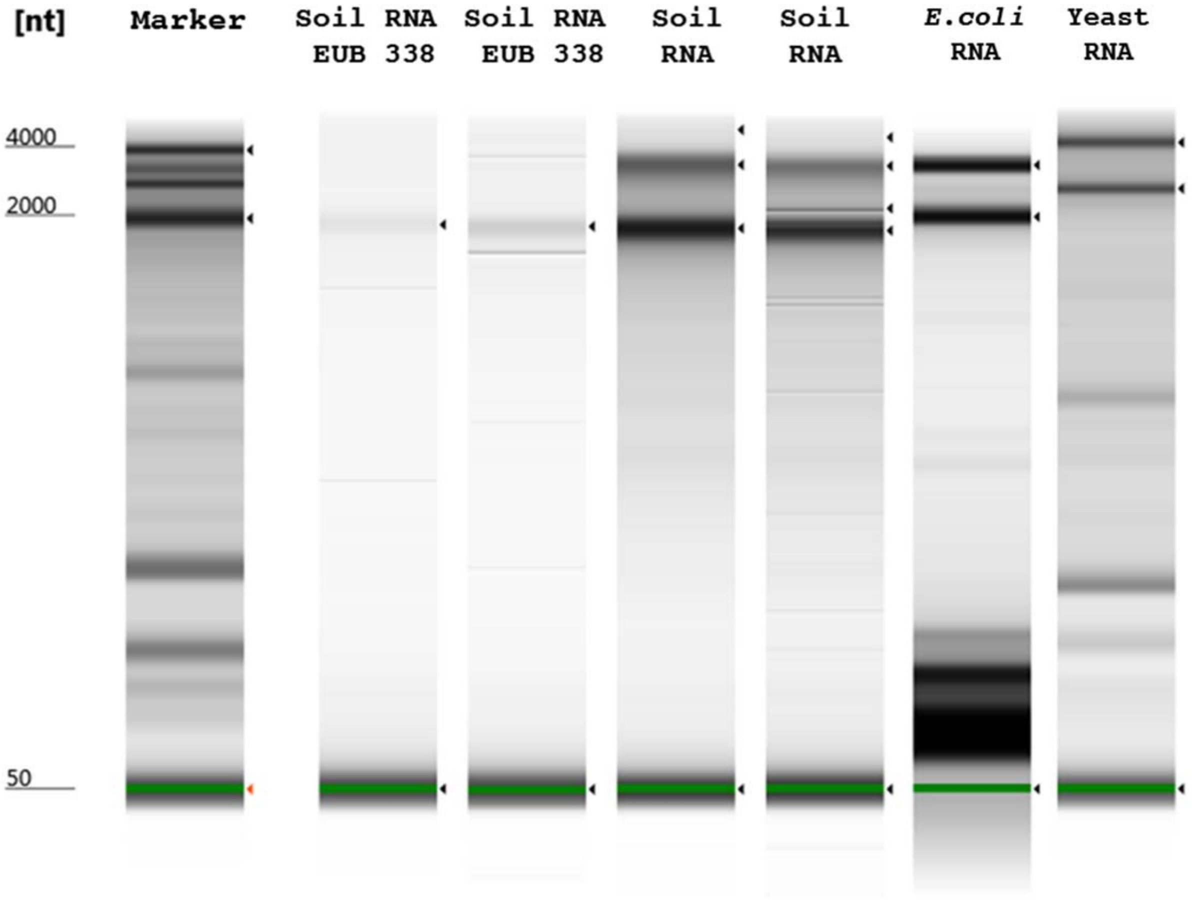

References:

Griffiths, R.I., Whiteley, A.S., O'Donnell, A.G., Bailey, M.J., 2000. Rapid method for coextraction of DNA and RNA from natural environments for analysis of ribosomal DNA- and rRNA-based microbial community composition. Applied and Environmental Microbiology, 66(12): 5488-5491.

Miyatake, T., MacGregor, B.J., Boschker, H.T.S., 2009. Linking Microbial Community Function to Phylogeny of Sulfate-Reducing Deltaproteobacteria in Marine Sediments by Combining Stable Isotope Probing with Magnetic-Bead Capture Hybridization of 16S rRNA. Applied and Environmental Microbiology, 75(15): 4927-4935.
